# A Rapid Review of Mental Health Training Programs for School Nurses

**DOI:** 10.1177/10598405241277798

**Published:** 2024-09-12

**Authors:** Cassidie S. Thomas, Tiffany K. Nielsen, Nakia C. Best

**Affiliations:** 1 Sue & Bill Gross School of Nursing, 8788 The University of California, Irvine, CA, USA

**Keywords:** school nurse, reviews, student mental health, school nurse continuing education

## Abstract

There is an urgent need for improved school-based mental health services to address students’ increasing mental health needs. School nurses are often at the frontlines of youth mental health, but report feeling unprepared to manage student needs due to limited training. We conducted a rapid review to identify evidence-based mental health educational interventions for school nurses and evaluate program characteristics. Eleven studies met the inclusion criteria. While the literature evaluating mental health training programs for school nurses is limited, it suggests that training may improve school nurse knowledge, confidence, and preparedness to address student mental health needs and improve the management of student mental health. Additionally, it highlighted the importance of support, resources, and policies that foster mental health promotion. Future research should focus on obtaining a current assessment of school nurse mental health education needs, evaluating existing interventions, and developing more evidence-based mental health training programs for school nurses.

Child and adolescent mental health is of increasing concern, with diagnoses such as anxiety, depression, and attention deficit hyperactivity disorder (ADHD), among other mental health conditions, impacting youth early in life. Data from the 2021 Youth Risk Behavior Survey (YRBS) showed increasing levels of poor mental health among adolescents, including persistent feelings of hopelessness or sadness and suicidal ideation (CDC, 2021). With nearly 50% of adolescents having been diagnosed with a mental health condition and nearly 1 in 5 children ages two to eight years having been diagnosed with a behavioral, mental, or developmental disorder, youth mental health must be prioritized to address this growing crisis (Cree et al., 2018; National Institute of Mental Health [NIMH], 2023).

There is an urgent need for improved school-based mental health services, given the delays in mental health care initiation and limited mental health resources that result from an under-staffed and overburdened youth mental health workforce in the United States (Kubik & Maughan, 2022; Smith & Payne, 2024). Schools have historically been critical in providing youth mental health services led by school counselors, psychologists, and social workers (Kubik & Maughan, 2022). In addition to these mental health professionals, school nurses are often at the frontlines contributing to students’ mental health care in schools (Bohnenkamp et al., 2015; Kubik & Maughan, 2022; NASN, 2021). However, two recent integrative reviews of school nurses’ role in student mental health showed that they are seldom actively engaged in delivering these services (Hoskote et al., 2023; Kaskoun & McCabe, 2022). While student mental health was identified as a school nursing priority in both integrative reviews, limited continuing education was identified as a significant barrier to their ability to effectively contribute to school-based mental and behavioral health (SBMH) teams, highlighting a need for professional development in this domain.

Hoskote et al. (2023) and Kaskoun and McCabe (2022) acknowledged the need to address this knowledge gap by implementing evidence-based professional development programs that prepare school nurses to address the mental health needs of students. However, they also noted the limitations in research related to school nurse mental health education needs, existing training programs, and the efficacy of these programs. Since the publication of both reviews, no synthesis of the literature with best practice recommendations for preparing school nurses for their role in youth mental health care has been published to our knowledge. Thus, this rapid review aims to identify existing evidence-based mental health training interventions for school nurses and evaluate the characteristics of effective programs.

## Background

Preparing individuals with the necessary education to identify and address mental health needs in community and healthcare settings is not a novel concept. Existing programs aimed at educating a general audience have been well-studied in the literature. One example of this approach is Mental Health First Aid (MHFA) training programs. Although not nursing-specific, this method has demonstrated improvements in participant mental health literacy among various populations of individuals, including healthcare providers, veterans, individuals living in rural communities, and youth (Costa et al., 2020; National Counsel for Mental Wellbeing, 2023; Ng et al., 2021). Systematic reviews of this MHFA approach by Costa et al. (2020) and Ng et al. (2021) highlight that MHFA programs, intended for a broad audience with diverse educational and professional backgrounds, might be too simple for healthcare professionals with higher baseline mental health knowledge, including school nurses.

Several resources and publications are available for school nurses that provide education and guidance on youth mental health assessment and management. For example, the National Association of School Nurses (NASN), the Centers for Disease Control and Prevention (CDC), and the American Academy of Pediatrics (AAP) each have webpages with links to additional resources dedicated to mental health in schools (AAP, 2024; CDC, 2023; NASN, 2024a, 2024b). Additionally, several academic journals regularly publish articles on youth mental health, including *NASN School Nurse*, *School Mental Health*, and *The Journal of School Nursing*. One example was a two-part article published by *NASN School Nurse* in 2021, providing education and resources for common youth mental health conditions (Perron et al., 2021a, 2021b). Although these resources and publications provide evidence-based educational materials, school nurses continue to report feeling unprepared to manage the mental health needs of students and may benefit from more organized training programs, skill development, and support aimed at increasing mental health knowledge (Bohnenkamp et al., 2019; Weaver et al., 2019).

Integrating support measures for the implementation of new knowledge into practice has long been valued in evidence-based practice (EBP) in nursing, where incorporating professional support, mentorship, and access to EBP resources plays a crucial role in the translation of knowledge into practice (Bianchi et al., 2018; Curtis et al., 2017). However, Bohnenkamp et al. (2015) found that most mental health training programs lack implementation support to help nurses apply new knowledge. Given that integrating support measures is a fundamental element of translating knowledge into practice, gaps in organized training programs and implementation support may be potential contributing factors to school nurses’ reduced confidence and preparedness to address students’ mental health needs.

Recent literature evaluating the role of school nurses in student mental health management and school nursing continuing education needs highlights the demand for mental health education by school nurses and the impact of training on their ability to address mental health needs. Morse et al. (2022) found that school nurses reported needing training on how to support students with mental health conditions and collaborate with educators, administration, and outside providers. In a study on school nurse continuing education needs by Jordan et al. (2024), school nurses also expressed a need for training in how to support students with mental health conditions such as anxiety, ADHD, substance use disorder (SUD), and behavioral challenges, as well as training on therapeutic communication. Finally, Kubik & Maughan (2022) underscored the need for role development for school nurses to practice within their full scope to meet student mental health needs through an action menu that included ongoing continuing education and a personal plan for continuous mental health skill development.

Evidence also suggests that a lack of continuing education may limit school nurses’ ability to contribute to mental health management through inadequate preparation and confidence (Hoskote et al., 2023; Kaskoun & McCabe, 2022). A recent NASN consensus document highlights the value of school nurses in SBMH teams through their potential to practice to the extent of their scope and apply key principles of NASN's *School Nursing Practice Framework* (2024a, 2024b) by conducting screening, managing referrals and care coordination, supporting crisis response and emergency preparedness, and providing direct mental health care to students, Nurses who lack the education and confidence to perform these skills may be limiting their ability to contribute to SBMH teams due to educational needs (NASN, 2023). Therefore, equipping school nurses with mental health education will better prepare them to fulfill their role as key members of SBMH teams, practicing to the extent of their scope and improving their ability to address the mental health needs of students (Turner & MacKay, 2015).

## Methods

### Design

**Figure 1.**
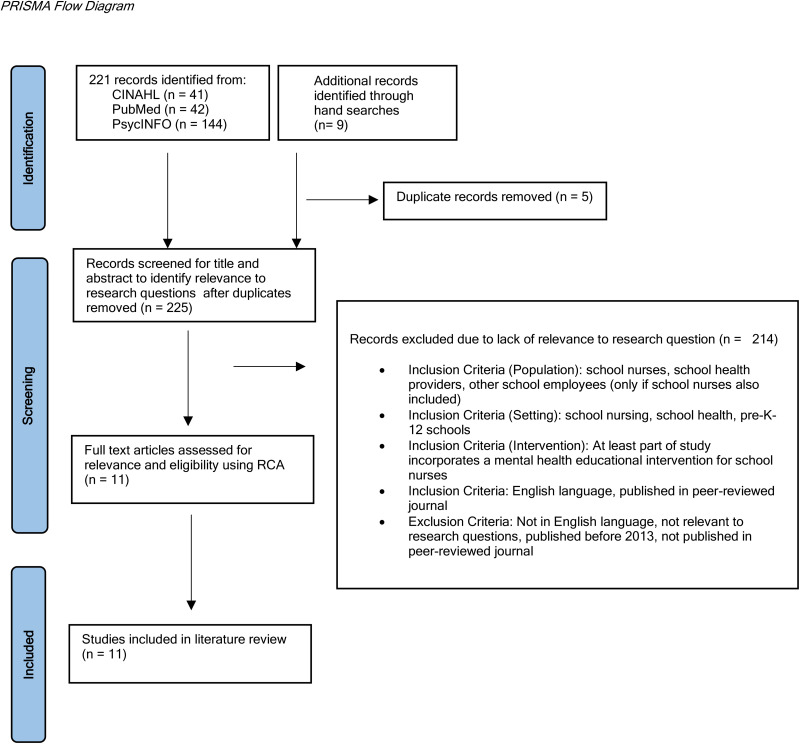
PRISMA flow diagram.

A rapid review of the literature was conducted according to Preferred Reporting Items for Systematic Reviews and Meta-Analyses (PRISMA) guidelines (See Figure 1; Page et al., 2021). The rapid review methodology was selected to capture published literature within the last ten years (Tricco et al., 2015). The guiding research questions for this review included:

1. What evidence-based mental health continuing education programs exist for school nurses?
2. What are the characteristics of effective mental health training programs for school nurses?

From: Page MJ, McKenzie JE, Bossuyt PM, Boutron I, Hoffmann TC, Mulrow CD, et al. (2021). The PRISMA 2020 statement: an updated guideline for reporting systematic reviews. BMJ, 372, 71.doi: 10.1136/bmj.n71.

### Search Methods

Literature published between 2013 and 2023 was searched in PubMed, PsycINFO, and CINAHL using search terms derived from the guiding research questions. Search strategies were discussed with an expert research librarian for Health Sciences and Nursing Science at the University of California, Irvine, before the search to identify keywords and techniques that best capture studies related to the research questions. Key search terms, including Medical Subject Headings (MeSH), were divided into three categories described below (See Supplementary Document for MeSH terms).

- population: school nurses, school health providers, nursery, preschool, primary, middle, intermediate, junior high and high school, and K-12 student
- intervention: mental health, mental health training intervention for health providers in schools, mental health inservice training, inservice training, workshop, mental health education, distance education, internet, professional development, youth mental health
- outcomes: mental health attitude measures, self-efficacy, preparedness

With this search strategy, 221 articles were identified from PubMed, PsycINFO, and CINAHL and nine articles published between 2013 and 2023 were identified through hand searches of the reference lists of studies included in this review and also of published literature in the *Journal of School Nursing*, *NASN* *School Nurse*, and the *Journal of School Health* using the following search terms*: mental health, continuing education, professional development, school nurse training, school nurse education, and school nurse intervention*.

### Inclusion and Exclusion Criteria

Five duplicates were removed after the initial literature search, and 225 articles were screened for relevance to the research questions through their titles and abstracts (Figure 1). Studies were included in the review if written in English, published in peer-reviewed journals, included school nurses working in pre-K-12 schools, and included mental health education for school nurses as part of the study. Exclusion criteria included articles not written in English, focused on a population that did not include school nurses, or included an intervention unrelated to mental health.

Due to limited studies in which interventions were implemented exclusively in a school nursing population, inclusion criteria were expanded to include two studies with other school employee participants in addition to school nurses. These included teachers, guidance counselors, a chaplain, and a youth worker for one study (McAllister et al., 2019) and teachers, guidance counselors, principals, administrators, social workers, public health professionals, and youth health care coordinators for the other (Wei & Kutcher, 2014). Additionally, studies were included if the intervention aimed to improve any aspect of school nurse identification or management of student mental health needs, including comprehensive mental health educational interventions or those focused on a specific topic such as anxiety or depression. Studies in which mental health education for nurses was not the study's primary aim were also included as long as the intervention was described and the training outcomes were evaluated, given the limited published literature exclusively evaluating nurse mental health training outcomes following an educational intervention. Finally, one study aimed to address substance use disorder (SUD) in an adolescent population through the Screening, Brief Intervention, and Referral to Treatment (SBIRT) process (Bourgault & Etcher, 2022). The SBIRT process aims to screen for individuals at risk for SUD and initiate a brief intervention, which may include referring students to mental health providers or rehabilitation if indicated. Given that SUD is classified as a mental health disorder by the Diagnostic and Statistical Manual of Mental Disorders, fifth edition (DSM-5), this study was included in this rapid review (APA, 2013). At the end of this screening step and application of exclusion criteria, 214 articles were eliminated, leaving eleven eligible studies to proceed with to the next stage of the appraisal process.

### Critical Appraisal

Rapid criticalappraisal (RCA) is a crucial component of the evidence-based practice process that helps to quickly evaluate the relevance, validity, and applicability of research evidence to practice. For this review, the authors used Melnyk & Fineout-Overholt's (2023) RCA Checklists to review each study. Once eligible studies were identified by reviewing their titles and abstracts, full texts were reviewed, and RCAs were performed by two of the authors of this review on each of the eleven studies based on the checklist associated with their level of evidence (LOE) (Melnyk & Fineout-Overholt, 2023). Using Melnyk & Fineout-Overholt's RCA Checklists, each study was assessed and ranked on a Low, Medium, and High scale for overall quality and utility for addressing the research questions (2023). This appraisal included reviewing each study's sampling methods, study design, data analysis, ethical considerations, transferability, limitations, and applicability in the practice setting of interest. All studies reviewed were ranked either Medium (n = 5) or High (n = 6) for overall quality in the RCA checklists. Levels of evidence ranged from II to VI on Melnyk & Fineout-Overholt's *Hierarchy of Evidence* (2023; See Table 1).

**Table 1.** Study Design Quality and Characteristics.

Source	1	2	3	4	5	6	7	8	9	10	11
Author & Year	McAllister et al. (2019)	Weaver et al. (2019)	Haddad et al. (2018)	Bohnenkamp et al. (2022)	Wei & Kutcher (2014)	Doi et al. (2018)	Muggeo et al. (2017)	Ginsburg et al. (2021)	Bourgault & Etcher (2022)	Allison et al. (2014)	Al-Yateem et al. (2023)
Training	*MHP Training*	*MH-TIPS*	*QUEST*	*MH-TIPS*	*Go-To Educator*	*New Role for SNs*	*CALM*	*CALM & CALM-R*	*SBIRT and S2BI Tool*	*PHQ-9 and SCARED Tools*	*MH Training*
LOE	III	III	II	III	III	VI	III	II	IV	III	VI
RCA Quality Rating	H	M	H	H	M	H	M	H	M	M	H
Sample Size	n = 27	n = 34	n = 146	n = 1282	n = 134	n = 27	n = 9 nurse; n = 11 student	n = 30 nurse; n = 54 student	n = 6	n = 6	n = 15

*Note*. LOE : Level of Evidence-based on Melnyk & Fineout-Overholt's Hierarchy of Evidence (2023); M : Medium; H : High; RCA: Rapid Critical Appraisal Checklist (Melnyk & Fineout-Overholt, 2023); Level of Evidence: I = systematic review; II = randomized controlled trial; III = controlled trial without randomization; IV = case-control or cohort study; V = systematic review of qualitative or descriptive studies; VI = qualitative or descriptive study; VII = expert opinion or consensus. Please note- Doi et al. is an evaluation of a program to refocus the school nurse role, details of the program can be found in the Scottish Government documents in the reference section of this review

### Data Extraction and Synthesis

After completing the RCA checklists for all eleven studies, each was reviewed for common themes highlighted in Tables 1 and 2. Table 1 provides an overview of study characteristics. Table 2 outlines the development of the synthesis stage, as themes were identified among eligible studies. These themes were grouped into the following categories: training characteristics, post-intervention outcomes for nurses, post-intervention outcomes for students, and internal support, policies, and resources. Finally, frequency distributions were constructed to demonstrate the occurrence of each theme and how it was expressed.

**Table 2.** Training Characteristics and Outcomes.

Variable	Frequency	Sources
**Curriculum Scope**		
**Comprehensive**	5	1,2,4,5, 11
**Condition-Specific**	5	3,7,8,9,10
**Partial**	1	6
**Screening Tool Role in Study**		
**Tool, Program, or Screening-Based Training**	4	7,8,9,10
**Tools & Screening included, but not the primary focus**	4	2,3,4,6
**Screening Tools not discussed**	3	1,5,11
**Educational Mode**		
**Online**	2	2,4
**In-Person**	9	1,3,5,6, 7,8,9,10,11
**Educational Approaches**		
**Didactic/In-Class Instructional Videos**	8	2, 3, 4, 7, 8, 9, 10, 11
**Group-Based**	2	1, 11
**Interactive**	3	1, 3, 11
**Role Play/Practice**	3	7, 9, 10
**Interactive Case Studies**	1	9
**Access to Additional Resources**	5	2, 3, 4, 7, 8
**Group Discussion**	1	9
**Post-Intervention Outcomes for Nurses and Students**		
**Nurse Confidence, Self-Efficacy, Empowerment, Preparedness**	7	1,2,4,6,9, 10,11
**Nurse Knowledge**	5	3,5,6, 9, 11
**Nurse Attitudes Toward Topic, Feasibility of Application of Knowledge, Training Satisfaction**	6	1,5,7, 9,11
**Nurse Outcomes Related to the Application of a Tool, Intervention, or New Approach**	3	3,4,6
**Improved Student Outcomes: Nurse Communication, Referrals, Symptoms, Functioning**	4	4, 7,8,10

*Note*. Access to Additional Resources: Includes any written resources, handouts in class setting, or visual or auditory digital resources.

## Results

The themes below emerged among the eleven studies included during the synthesis stage of this rapid review process. Study characteristics and sources can be found in Table 1. Themes were synthesized in Table 2.

### Design and Demographics

Of the eleven studies included in this literature review, six reported results from in the United States (Allison et al., 2014; Bohnenkamp et al., 2022; Bourgault & Etcher, 2022; Ginsburg et al., 2021; Muggeo et al., 2017; Weaver et al., 2019), one in Australia (McAllister et al., 2019), one in England (Haddad et al., 2018), one in Canada (Wei & Kutcher, 2014), one in Scotland (Doi et al., 2018), and one in the United Arab Emirates (Al-Yateem et al., 2023). Four of the studies employed quasi-experimental methodologies (Bohnenkamp et al., 2022; Muggeo et al., 2017; Weaver et al., 2019; Wei & Kutcher, 2014), one qualitative design (Al-Yateem et al., 2023), two randomized controlled methodologies (Ginsburg et al., 2021; Haddad et al., 2018), two mixed-methods using a realist evaluation approach (Doi et al., 2018; McAllister et al., 2019), and two quality improvement projects (Allison et al., 2014; Bourgault & Etcher, 2022). The sample size ranged from 6 to 1,282 participants, with the largest sample being associated with a web-based intervention (Bohnenkamp et al., 2022). All studies evaluated educational interventions in a population of school nurses; two studies also included additional school employees in their programs, including teachers, school counselors, administrators, and other school employees (McAllister et al., 2019; Wei & Kutcher, 2014).

### Training Characteristics

Each study was evaluated for the content and structure of the intervention, educational delivery mode, approach to teaching participants, and implementation support provided for nurses. Frequency distributions for training characteristics can be found in Table 2 to demonstrate the prevalence of various training approaches, including the scope of the curriculum, the role of screening tools in the study, educational delivery mode, educational approaches, and post-intervention outcomes for nurses and students.

#### Content and Structure

The curriculum used in each intervention was evaluated to determine its breadth and grouped into the following categories: comprehensive, partial, and single-subject. Comprehensive curriculum was defined as a curriculum covering an array of mental health topics to give participants a broad base of mental health knowledge aimed at equipping them to address a variety of mental health scenarios. Partial mental health curriculum was defined as a curriculum covering more than one topic but not aimed to give a broad mental health knowledge base and focused on a select set of conditions. Finally, single-subject curriculum included interventions focused on only one mental health condition, such as anxiety or depression.

Of the studies included, five implemented an intervention that implemented comprehensive mental health curriculum (Al-Yateem et al., 2023; Bohnenkamp et al., 2022; McAllister et al., 2019; Weaver et al., 2019; Wei & Kutcher, 2014), five covered single-subject mental health content for either anxiety (Allison et al., 2014; Ginsburg et al., 2021; Muggeo et al., 2017), depression (Haddad et al., 2018), or substance use disorder (Bourgault & Etcher, 2022), and one focused on the identification of potential mental health concerns using the Strengths and Difficulties Questionnaire (SDQ), followed by referral pathways for students and, thus, was categorized as partial mental health curriculum (Doi et al., 2018).

Four studies evaluated the application of a condition-specific mental health tool or screening instrument by school nurses following a training intervention (Allison et al., 2014; Bourgault & Etcher, 2022; Ginsburg et al., 2021; Muggeo et al., 2017) and four incorporated elements of education related to mental health screening or tools, although this was the not primary focus of the study (Bohnenkamp et al., 2022; Doi et al., 2018; Haddad et al., 2018; Weaver et al., 2019).

Two studies examined comprehensive mental health curriculum and took place over one day to two weeks (Al-Yateem et al., 2023; Bohnenkamp et al., 2022; McAllister et al., 2019; Weaver et al., 2019; Wei & Kutcher, 2014). The two studies with single-subject mental health curriculum and one with partial curriculum took place over two to three hours (Allison et al., 2014; Bourgault & Etcher, 2022; Doi et al., 2018).

#### Education Delivery Mode

The two educational delivery modes identified in the studies were web-based training modules and in-person training. Web-based training consisted of nurses completing training online, asynchronously, and at their own pace, while in-person training involved groups of participants engaging in instructor-led training at a set time in a classroom setting. Of the studies included, two implemented the same self-paced, web-based mental health educational intervention (Bohnenkamp et al., 2022; Weaver et al., 2019), although Weaver et al. (2019) hosted web-based training at an on-site location during a pre-scheduled meeting time. Nine studies implemented in-person interventions (Allison et al., 2014; Al-Yateem et al., 2023; Bourgault & Etcher, 2022; Doi et al., 2018; Ginsburg et al., 2021; Haddad et al., 2018; McAllister et al., 2019; Muggeo et al., 2017; Wei & Kutcher, 2014). The two web-based studies utilized the Mental Health Training Intervention for Health Providers in Schools (MH-TIPS), an evidence-based, comprehensive mental health education program created by the University of Maryland School of Medicine in partnership with the National Association of School Nurses and the Center for Mental Health Services in Pediatric Primary Care at the Johns Hopkins Bloomberg School of Public Health (Bohnenkamp et al., 2019). This training program was developed using a data-driven process and later modified through a multilevel stakeholder-driven follow-up study to refine its educational content and approaches and improve contextual relevance and uptake among populations of school nurses. The comprehensive curriculum covered in MH-TIPS includes education on referrals and resource mapping, techniques to support student mental health needs, identification and assessment, crisis response, mental health interventions, and information about psychotropic medication. Both published studies evaluating MH-TIPS were included in this review, and results showed post-intervention improvements in school nurses’ perceived preparedness to address mental health needs, similar to the post-intervention improvements in confidence, empowerment, and preparedness of in-person interventions (Allison et al., 2014; Al-Yateem et al., 2023; Bohnenkamp et al., 2022; Doi et al., 2018; Weaver et al., 2019). See Table 2 for an overview of educational delivery modes.

#### Educational Approach

Educational approaches across studies incorporated multi-modal training structures with elements of didactic teaching through lectures and instructional videos, role play, case studies, group-based discussion, and other interactive approaches. Access to written and digital resources was also common in training programs. Of the eleven studies reviewed, eight incorporated at least some didactic teaching in their training (Allison et al., 2014; Al-Yateem et al., 2023; Bohnenkamp et al., 2022; Bourgault & Etcher, 2022; Ginsburg et al., 2021; Haddad et al., 2018; McAllister et al., 2019; Muggeo et al., 2017; Weaver et al., 2019). Three studies implemented interactive training (Al-Yateem et al., 2023; Haddad et al., 2018; McAllister et al., 2019). Role play and opportunities to practice new skills were included in three studies (Allison et al., 2014; Bourgault & Etcher, 2022; Muggeo et al., 2017), and interactive case studies were included in one (Bourgault & Etcher, 2022). Access to additional on-demand resources was an approach utilized in five studies and included training manuals, handouts, instructional videos and audio files, relevant resource links, and resource libraries for participants (Bohnenkamp et al., 2022; Ginsburg et al., 2021; Haddad et al., 2018; Muggeo et al., 2017; Weaver et al., 2019). Lastly, one study included group discussion as a component of training (Bourgault & Etcher, 2022). A breakdown of training elements can be found in Table 2.

#### Ongoing Support

Among a majority of the studies, access to educational resources or ongoing support was acknowledged to be a characteristic of mental health training that could help with the translation of new knowledge into practice (Al-Yateem et al., 2023; Bohnenkamp et al., 2022; Bourgault & Etcher, 2022; Doi et al., 2018; Ginsburg et al., 2021; Muggeo et al., 2017; Weaver et al., 2019). However, among the studies that included access to educational resources or ongoing support, there was substantial variability in their approach to implementing these support measures for participants.

Al-Yateem et al. (2023) implemented a unique approach to ongoing support and feedback for participants through a training program that unfolded across a two-week time frame where participants engaged in educational sessions daily at the end of the workday. Through thematic analysis of post-intervention written reflections and interview data, participants expressed the ability to apply new knowledge in practice and receive feedback and support from trainers, which allowed for the simultaneous development and application of new knowledge.

In the study conducted by Doi et al. (2018), participants were given time between training to apply knowledge and skills learned through the training in their workplace. However, training lasted two days, so participants had only one scheduled opportunity for trainer feedback and reflection in the classroom following application. The authors of this study also attempted to initiate a preceptorship program in one location, a values-based reflection practice at another site, and monthly follow-up sessions for participants to reinforce learning and allow feedback. These support measures were eventually discontinued, citing trainer and nurse scheduling conflicts as the primary barrier. Despite various approaches to offering ongoing support for nurses, some suggested that ongoing training and support in mental health pathways would help aid in the application of new knowledge in the post-intervention qualitative interview data.

Two studies provided nurses with the option of expert consultation with a licensed clinical psychologist as nurses applied a new student mental health intervention into practice (Ginsburg et al., 2021; Muggeo et al., 2017). This support was available via email or phone to increase nurses’ access but was discontinued post-intervention due to the expense associated with maintaining this resource (Muggeo et al., 2017). Both studies also provided nurses access to materials such as training videos, handouts, and an intervention manual and acknowledged the importance of ongoing performance feedback and clinical supervision as nurses implemented new knowledge and skills (Ginsburg et al., 2021; Muggeo et al., 2017).

The two studies that evaluated the impacts of web-based interventions had self-paced training models (Bohnenkamp et al., 2022; Weaver et al., 2019). Given the flexibility of this training approach, nurses may have had the opportunity to apply knowledge in their workplaces throughout the training process. However, this was not built into the training structure or monitored in either study. Participants also had access to all educational and support materials within the modules. However, there was no access to trainer feedback with this particular training structure. Lastly, one study recommended the integration of a follow-up meeting two weeks post-implementation of a new screening tool to reinforce education and address any concerns in future studies (Bourgault & Etcher, 2022).

### Post-Intervention Outcomes for Nurses

School nurse training programs were associated with improved post-intervention nurse confidence, self-efficacy, empowerment, and preparedness. Additionally, mental health training programs were associated with post-intervention improvements in nurses’ mental health knowledge, attitudes toward mental health, training satisfaction, and perspectives on the feasibility of applying new skills into practice. Frequency distributions of these nurse outcomes are listed in Table 2.

#### Confidence

The thematic analysis of qualitative data reported that training improved nurses’ confidence in initiating student communication about mental health concerns, and nurses reported improvements in their practice (Al-Yateem et al., 2023). Another study reported a significant improvement in confidence and nurse preparedness to facilitate a mental health program (p = <.001; McAllister et al., 2019). Self-efficacy related to the use of the Screening, Brief Intervention, and Referral to Treatment (SBIRT) process resulted in improvements in communication with students, identification of students meeting criteria for screening, and student referrals (p = <.05; Bourgault & Etcher, 2022).

#### Empowerment

Qualitative data from Al-Yateem et al. (2023) found that training empowered nurses to use new mental health knowledge, expanded their practice to care for students, and made them feel like their role in mental health care was significant to their school communities. Results from a qualitative study by Allison et al. (2014) revealed that implementing mental health screening tools for students empowered nurses to initiate and improve the quality of conversations about mental health with students and their families.

#### Preparedness

Another nurse outcome assessed in some of the studies was participant perceptions of preparedness to address mental health needs, including the identification of mental health concerns, communication with students and families about mental health concerns, the application of brief mental health interventions for students, and the initiation mental health referrals for students. Improvements in perceived preparedness in each of these areas were used in each of these studies as a potential indicator of improvements in mental health post-intervention management, although none of the studies explicitly studied this outcome (Bohnenkamp et al., 2022; Doi et al., 2018; Weaver et al., 2019).

Perceived preparedness to address mental health needs was evaluated in two of the studies via pre- and post-intervention Likert-type scales ranging from 1 = very low to 5 = very high (Bohnenkamp et al., 2022) and 0 = very low to 4 = very high (Weaver et al., 2019), while one study evaluated this outcome via post-intervention focus group data (Doi et al., 2018). Training in mental health was associated with improvements in overall nurse preparedness to address student mental health needs (p = <.001; Bohnenkamp et al., 2022) and self-rated preparedness to identify psychological distress in students and intervene appropriately (p = <.05; Weaver et al., 2019). Increased mental health training time was associated with improved nurse self-perceived preparedness to motivate students to seek help (p = .17) and nurse preparedness to conduct a brief mental health intervention (p = .028; Bohnenkamp et al., 2022). Focus group data from Doi et al. (2018) also suggested that nurses gained preparedness to refer students with complex mental health concerns through improvements in self-reported preparedness. Still, nurses expressed the need for further training on less complicated mental health needs.

#### Knowledge

Mental health education was associated with post-training improvements in school nurse knowledge of the mental health topics covered, including foundational mental health knowledge as well as specific knowledge about depression and SUD. Improvements in post-intervention mental health knowledge in signs, symptoms, onset, and causes of mental health disorders, mental health tools and assessment techniques, and best practices for referral and communication were measured in one study via a 30-question survey taken by participants pre- and post-intervention (p = <.0001; Wei & Kutcher, 2014). To measure participant depression knowledge at baseline and three and nine months post-intervention, one study used the QUEST knowledge measure, an evidence-based depression knowledge measurement tool created by the authors in a prior study (Haddad & Tylee, 2013; Haddad et al., 2018). The QUEST knowledge measure is a 24-item survey measuring participant knowledge of the clinical features and overall condition description of depression, predictors and risk factors, and treatment, management, and referral criteria. Using the QUEST knowledge measure, participants in this study demonstrated significant improvements in depression knowledge at three months post-intervention (p = <.001) and nine months post-intervention (p = <.001).

Substance use disorder (SUD) knowledge improvements were measured as part of a 10-question Likert-type scale at baseline, after SUD and SBIRT training, and six weeks post-intervention (p = <.003; Bourgault & Etcher, 2022). Self-reported improvements in general mental health knowledge were also highlighted through thematic analysis in post-intervention focus groups and written reflections (Al-Yateem et al., 2023; Doi et al., 2018).

#### Attitudes, Feasibility, and Satisfaction

Mental health education was associated with improved attitudes toward mental health (p = <.0001; Wei & Kutcher, 2014) and improved acceptance of new approaches to mental health needs in practice (p = <.001; Bourgault & Etcher, 2022). Qualitative thematic analysis revealed that participants found the mental health training relevant and important to school nursing practice (Al-Yateem et al., 2023; McAllister et al., 2019). Additionally, post-intervention satisfaction ratings of training were high for one study that evaluated the training and implementation of a nurse-led intervention for child anxiety through a program called the Child Anxiety Learning Modules (CALM; Muggeo et al., 2017). Training satisfaction for this study was measured using a 7-point Likert-type scale ranging from 1 (not at all) to 7 (very much), and mean scores for each survey question were calculated. Post-intervention training mean satisfaction ratings by participants (n = 9) were high in the areas of training usefulness (6.56), nurse preparedness (6.88), and overall satisfaction (7).

#### Application of Knowledge

School nurses reported improved application of new tools or knowledge to practice following mental health training. These measurements were evaluated separately from outcomes that were linked to student outcomes. Bohnenkamp et al. (2022) measured the application of knowledge 30 days post-intervention, with the following percentages of respondents reporting that they used the following training elements at least sometimes: common mental health skills (76.19%), psychotropic medication knowledge (51.47%), student support (77.75%), mental health identification and assessment (77.40%), student referrals and resource mapping (71.01%), mental health crisis response and safety assessments (64.18%). Doi et al. (2018) reported improvements in the identification of children's mental health needs by school nurses post-education, as demonstrated by 68% of all referrals being for mental health needs, with the remaining 32% being related to substance abuse, domestic abuse, housing, transitions, or caregiver concerns, and youth justice referrals. Although not statistically significant, Haddad et al. (2018) reported improved participant ability to identify cases representing depression in vignette-based depression judgments between groups. However, they did find that training was associated with a statistically significant improvement in school nurses’ ability to identify cases that did not represent individuals with depression in vignette-based depression judgments compared with the control group (p = .039).

### Post-Intervention Outcomes for Students

Mental health education was also linked to improvements in student symptoms of anxiety, concentration, global functioning, and student referrals, as well as increases in the number of students approached about mental health concerns by the school nurse (Bohnenkamp et al., 2022; Ginsburg et al., 2021; Muggeo et al., 2017).

Results from Muggeo et al. (2017) found that by training school nurses to deliver a student anxiety intervention program and then implementing the intervention in a student population, students experienced a reduction in self-reported student anxiety (p = .004) and parent-reported student anxiety (p = .001). These outcomes were measured using the Screen for Child Anxiety-Related Emotional Disorders (SCARED) tool designed to measure child- and parent-reported childhood anxiety (Birmaher et al., 1997, 1999). Students in this study also experienced reductions in parent-reported somatic symptoms of student anxiety (p = .016). These outcomes were measured using the Child Somatization Inventory (CSI-24), a scale measuring parent-and child-reported somatic complaints (Walker et al., 2009). Improvements were also noted in student global functioning (p = .003) measured by the Children's Global Assessment Scale (CGAS), a rating scale completed by an evaluator that serves as an indication of a child's global functioning over the past month (Shaffer et al., 1983). Finally, a reduction in teacher-reported concentration difficulties was also noted in this study (p = .02) using the Teacher Observation of Classroom Adaptation Checklist (Koth et al., 2009).

Ginsburg et al. (2021) continued to evaluate the student anxiety intervention Muggeo et al. (2017), adding a comparison group focused on relaxation only. The authors reported improvements in student global anxiety severity ratings from baseline to three months post-intervention in 58.8% of students in the intervention group and 34.4% in the comparison group (Ginsburg et al., 2021). These improvements were measured using the Clinical Global Impression- Improvement (CGI-I) rating, which measures clinical improvements in anxiety from baseline (Walkup et al., 2008). Across the board, both groups in this study showed improvement in anxiety symptoms measured by the CSI-24 and improved functioning measured by the Child Anxiety-Impact Scale (CAIS; Langley et al., 2014), the Children's Automatic Thoughts Scale (Schniering & Rapee, 2002) and the Behavioral Avoidance Scale (Ginsburg et al., 2021).

Finally, training was also associated with nurse-reported increases in the number of students they approached due to mental health concerns, with pre-training average weekly student rates of 2.67 and post-intervention rates of 3.5 (Bohnenkamp et al., 2022). For frequency distributions related to student outcomes, see Table 2.

## Discussion

While this rapid review aimed to identify existing evidence-based mental health training interventions for school nurses and evaluate characteristics of effective programs, we found limited published literature in this domain. Although eleven studies were included in the review, only nine unique training programs were identified, given that two of the interventions were repeated in subsequent studies that were also included in this review (Bohnenkamp et al., 2022; Ginsburg et al., 2021; Muggeo et al., 2017; Weaver et al., 2019). Moreover, the mental health conditions covered in training varied, with some interventions only covering a specific topic (i.e., depression only), while others provided participants with a broad mental health foundation. Bohnenkamp et al. (2015) also found a deficit of comprehensive, evidence-based mental health training programs for school nurses and limited literature evaluating nurse and student training outcomes. Regardless, the authors highlighted the potential benefits of training on identifying and managing student mental health needs. Kaskoun and McCabe (2022) drew a similar connection between training and nurse confidence, suggesting that improved nurse confidence from training may impact school nurses’ ability to support student mental health needs. These findings share some consistencies with those found in this rapid review, which suggests that training may improve school nurse knowledge, confidence, empowerment, and preparedness to address student mental health needs.

Additionally, results from this review showed that these improvements for school nurses may also positively impact student outcomes related to the identification of mental health concerns, initiation of appropriate referrals, reduction of student symptoms, and improvements in the overall management of student mental health. However, we cannot infer a cause-and-effect relationship between training and these outcomes due to the limited literature on this subject. Despite these limitations, these studies gave insight into training features and approaches that may help inform future education and research initiatives in school nurse mental health continuing education.

One key finding of this rapid review was the value of providing ongoing support and access to resources for school nurses to aid in the application of new knowledge. Studies supported participants through a variety of approaches, including providing access to consultation, preceptors, opportunities to receive feedback from trainers, follow-up sessions, as well as training videos, handouts, and manuals (Al-Yateem et al., 2023; Bohnenkamp et al., 2022; Bourgault & Etcher, 2022; Doi et al., 2018; Ginsburg et al., 2021; Muggeo et al., 2017; Weaver et al., 2019). This aligns with the fundamental elements of EBP, where professional support, mentorship, and access to educational resources aid in translating new knowledge and skills into practice (Bianchi et al., 2018; Curtis et al., 2017). In previous studies, school nurses have benefited from incorporating these support measures, helping improve, apply, and sustain knowledge related to student conditions such as asthma (Trivedi et al., 2018) and obesity (Tucker & Lanningham-Foster, 2015). Although findings from this rapid review cannot conclude that these support measures led to improved student or nurse outcomes, they align with best practice recommendations for EBP. Thus, future program development for school nurse mental health training should consider incorporating ongoing support and access to resources for participants.

This rapid review also highlighted that mental health professional development for school nurses can only be fully implemented in the presence of administrative support, internal policies supporting mental health efforts, adequate space and staffing, and, importantly, valuing mental health care for students (Allison et al., 2014; Doi et al., 2018; Ginsburg et al., 2021; Haddad et al., 2018; Muggeo et al., 2017). To address student mental health from an internal school policy perspective, one study recommended that student health history forms be updated to incorporate social, emotional, and behavioral health information to demonstrate the prioritization of student physical and emotional health (Allison et al., 2014). In addition to modifying school health forms, internal policy changes that implemented specific student referral pathways for school nurses gave nurses the administrative support and structure to guide student mental health management and streamline referrals (Doi et al., 2018) These approaches are in alignment with recommendations by the integrative review by Hoskote et al. (2023), who identified a need for the collaboration of schools, districts, and community partners to create evidence-based policies and procedures aimed at student mental health promotion both on and off-campus. However, Kaskoun and McCabe (2022) assert that mental health policies should also address school nurse mental health training requirements that aim to improve the overall preparedness of the school nursing workforce to address the increasing mental health needs of students.

In addition to the value of implementing policies and procedures that promote student mental health, barriers to mental health promotion efforts, such as school nurse staffing, space, and workload issues were also identified as important considerations. Ginsburg et al. (2021) stated that school nurses’ competing demands and time constraints could have hindered their time spent on a mental health intervention and impacted student access. This sentiment was echoed by Haddad et al. (2018), who highlighted the importance of ensuring adequate staffing to support school nurses in their expanding roles. Lastly, interview data from Muggeo et al. (2017) identified school nurse-designated space and time as limiting factors to implementing school-nurse-led student mental health interventions, stating that administrative support of these resources is critical to school nurses being able to address student mental health needs without interruption or distraction. These potential barriers to mental health initiatives suggest staffing, space, and other workplace concerns may also be essential factors in school nurse management of student mental health. Kubik and Maughan (2022) recognized the importance of addressing these potential barriers, urging school nurses to advocate for appropriate staffing ratios and contribute to efforts to obtain sustainable funding for mental health services in addition to school nurse training and policy development. Therefore, in addition to supporting continuing education for school nurses, schools must aim to address mental health promotion from an administrative, ensuring that resources, staffing, and policies are aligned in the best interest of students’ mental health needs.

However, even with sufficient support via policies, procedures, staffing, space, and funding, school nurses may be limited in their ability to address student mental health needs if they are not considered primary members of SBMH teams. One study in this review stated that not all school nurses view mental health interventions as their responsibility (Ginsburg et al., 2021). Evidence supports this statement, as nurses are not always considered part of the SBMH team in schools even though one in three visits to the school nurse by students is for a mental health reason; however, the importance of expanding the role of the school nurse in student mental health is widely recognized as a priority (Bohnenkamp et al., 2015; Hoskote et al., 2023; Kaskoun & McCabe, 2022; Kubik & Maughan, 2022). Kubik and Maughan (2022) recently published a call to action, urging school nurses and administrators to identify school nurses as members of SBMH teams to advance the role of school nurses in student mental health care. However, recognition as an SBMH team member must also be matched with the professional development necessary to confidently and effectively serve as members of these teams (Kaskoun & McCabe, 2022). As qualitative data in this review found that training improved school nurse confidence and sense of identity as key SBMH team members, expanding the role of the school nurse in student mental health management should also incorporate a focus on role development through continuing education (Al-Yateem et al., 2023).

## Limitations

This review focused only on literature published within the last ten years using key search terms derived from the research questions. Thus, it is possible that published studies that could have contributed to this review were missed. To address this potential limitation, an expert research librarian for Health Sciences and Nursing Science was consulted to discuss search strategies, keywords, and other techniques that would best capture relevant research. Although this search strategy resulted in the variability of study designs and training content, it aimed to capture the most current and relevant literature on this topic to meet the evolving needs and complexities of student mental health.

## Implications for School Nursing Practice and Conclusion

School nurses are well-positioned to care for students’ mental and behavioral health needs (NASN, 2021). They are not only on the frontlines of student mental health needs but have the potential to approach mental health care from a holistic perspective by providing direct care, conducting screenings, managing referrals and care coordination, and supporting and responding to mental health emergencies, making them critical members of SBMH teams (NASN, 2023). Using the key principles from NASN's *School Nursing Practice Framework* (2024a, 2024b) of standards of practice, care coordination, leadership, and community/public health, school nurses can improve the provision of school-based mental health care by expanding their role and practicing to their full scope (Kaskoun & McCabe, 2021). However, school nurses must be equipped with evidence-based education that prepares them to engage in student mental health care confidently.

Although this review aimed to begin the work of identifying and evaluating existing evidence-based mental health training programs for school nurses, we found limited literature on this topic. Thus, several areas require further development to understand the state of school nurse mental health preparation fully.

Among these areas requiring further development is a more in depth understanding of current school nurse mental health education gaps. Hoskote et al. (2023) highlighted the need for a thorough assessment of school nurse mental health continuing education gaps. In 2014, Bohnenkamp et al. (2015) distributed polls via NASN Weekly Electronic Digest, an e-news communication distributed to all NASN subscribers and members. These polls collected national data on school nurse mental health practices and ultimately informed the development of MH-TIPS, demonstrating the value of conducting a needs assessment to inform future mental health program development. Additionally, gaining an understanding of school nurse baseline mental health knowledge, information about engagement in previous training programs, and an understanding of the most commonly used approaches to mental health education that school nurses engage in would provide a better understanding of education gaps and awareness of mental health training programs or materials that may not be published in the literature.

In addition to conducting a needs assessment on education gaps and previous education, further research is needed to evaluate the efficacy of existing mental health educational interventions for school nurses. Results from this rapid review, as well as previous integrative reviews, have shed light on the limited published literature on this topic as well as the need for more data to inform best practice recommendations and future program development (Hoskote et al., 2023; Kaskoun & McCabe, 2022). However, this may also indicate that there is not just a gap in the literature on this topic but also in existing mental health programs for school nurses. Therefore, members of SBMH teams, school administrators, researchers, and community partners must continue to engage in collaborative efforts to promote mental health in schools, with a particular focus on the development of accessible, evidence-based mental health continuing education programs for school nurses as well as professional support, resources, policies, and values that promote the expanding role of school nurses in school-based mental health services. With these elements and using NASN's *School Nursing Practice Framework* (2024a, 2024b) to guide their practice, school nurses will have the necessary support and education to be critical SBMH team members and improve school-based mental health services.

## Supplemental Material

sj-docx-1-jsn-10.1177_10598405241277798 - Supplemental material for A Rapid Review of Mental Health Training Programs for School Nurses Supplemental material, sj-docx-1-jsn-10.1177_10598405241277798 for A Rapid Review of Mental Health Training Programs for School Nurses by Cassidie S. Thomas, Tiffany K. Nielsen and Nakia C. Best in The Journal of School Nursing
