# Supplementary material for: A Rapid Review of Mental Health Training Programs for School Nurses
Source: J Sch Nurs. 2024 Sep 12;41(1):158–71. doi: 10.1177/10598405241277798 (PMC11755978; doi:10.1177/10598405241277798)
Supplement: sj-docx-1-jsn-10.1177_10598405241277798 - Supplemental material for A Rapid Review of Mental Health Training Programs for School Nurses [file sj-docx-1-jsn-10.1177_10598405241277798.docx]

**Complete Search Strategy**

**PsycInfo**

MH-TIPS OR "Mental Health Training Intervention for Health Providers in Schools" OR MAINSUBJECT.EXACT("Mental Health Inservice Training") OR ("mental health" NEAR/3 (train* OR educat*))

AND

MAINSUBJECT.EXACT("Intermediate School Students") OR MAINSUBJECT.EXACT("Preschool Students") OR MAINSUBJECT.EXACT("Primary School Students") OR MAINSUBJECT.EXACT("Elementary School Students") OR MAINSUBJECT.EXACT("Middle School Students") OR MAINSUBJECT.EXACT("Nursery School Students") OR MAINSUBJECT.EXACT("High School Students") OR "k-12"

Limits: 2012 - 2023

MAINSUBJECT.EXACT("Professional Development"))

AND

MAINSUBJECT.EXACT("Youth Mental Health") OR MAINSUBJECT.EXACT("Mental Health") OR MAINSUBJECT.EXACT("Mental Health Attitude Measures")

AND

MAINSUBJECT.EXACT("Intermediate School Students") OR MAINSUBJECT.EXACT("Preschool Students") OR MAINSUBJECT.EXACT("Primary School Students") OR MAINSUBJECT.EXACT("Elementary School Students") OR MAINSUBJECT.EXACT("Middle School Students") OR MAINSUBJECT.EXACT("Nursery School Students") OR MAINSUBJECT.EXACT("High School Students") OR "k-12"

Limits: 2012 - 2023

**PubMed**

("School Nursing"[Mesh] OR "school nurs*") AND (MH-TIPS OR "Mental Health Training Intervention for Health Providers in Schools" OR "Mental Health/education"[Mesh] OR ("mental health"[tiab] AND (train*[tiab] OR educat*[tiab])

("School Nursing"[Mesh] OR "school nurs*") AND ("Inservice Training"[Mesh] OR orientation* OR workshop* OR "Professional Development" OR "Education, Nursing, Continuing"[Mesh] OR "continuing education" OR "professional development" OR inservice) AND ("Students"[Mesh:NoExp] OR "high school*" OR "secondary school*" OR "middle school*" OR "junior high school*" OR "elementary school*" OR "primary school*" OR "nursery school*" OR pre-school* OR "k-12")

("School Nursing"[Mesh] OR "school nurs*") AND ("Inservice Training"[Mesh] OR orientation* OR workshop* OR "Professional Development" OR "Education, Nursing, Continuing"[Mesh] OR "continuing education" OR "professional development" OR inservice) AND ("Students"[Mesh:NoExp] OR "high school*" OR "secondary school*" OR "middle school*" OR "junior high school*" OR "elementary school*" OR "primary school*" OR "nursery school*" OR pre-school* OR "k-12") AND ("School Mental Health Services"[Mesh] OR "Mental Health"[Mesh] OR "mental health"[tiab])

**CINAHL**

(MH "School Health Nursing") OR (school N3 nurs*)

AND

MH-TIPS OR "Mental Health Training Intervention for Health Providers in Schools" OR (MH "Mental Health/ED") OR ("mental health" N5 (train* OR educat*))

Limiters - Published Date: 20120101-; English Language

(MH "School Health Nursing") OR (school N3 nurs*)

AND

(MH "Inservice Training") OR (MH "Staff Development") OR (MH "Professional Development+") OR (MH "Education, Nursing, Continuing") OR (MH "Education, Continuing") OR "continuing education" OR "professional development" OR inservice

AND

(MH "Students, High School") OR (MH "Students, Middle School") OR (MH "Schools, Middle") OR (MH "Schools, Secondary") OR (MH "Schools, Nursery") OR "k-12"

AND

(MH "Mental Health") OR (MH "School Mental Health Services") OR "mental health"

(MH "School Health Nursing") OR (school N3 nurs*)

AND

(MH "Inservice Training") OR (MH "Staff Development") OR (MH "Professional Development+") OR (MH "Education, Nursing, Continuing") OR (MH "Education, Continuing") OR "continuing education" OR "professional development" OR inservice

AND

(MH "Mental Health") OR (MH "School Mental Health Services") OR "mental health" OR MH-TIPS OR "Mental Health Training Intervention for Health Providers in Schools" OR (MH "Mental Health/ED")

AND

(MH "Students, High School") OR (MH "Students, Middle School") OR (MH "Schools, Middle") OR (MH "Schools, Secondary") OR (MH "Schools, Nursery") OR "k-12" OR student*

Narrow by Language: - english
